# Deficits in cholinergic neurotransmission and their clinical correlates in Parkinson’s disease

**DOI:** 10.1038/npjparkd.2016.1

**Published:** 2016-02-18

**Authors:** Santiago Perez-Lloret, Francisco J Barrantes

**Affiliations:** 1 Institute of Cardiologic Research, National Scientific and Research Council (ININCA-CONICET), Faculty of Medicine, University of Buenos Aires, Buenos Aires, Argentina; 2 Laboratory of Molecular Neurobiology, Institute for Biomedical Research, UCA-CONICET, Faculty of Medical Sciences, Buenos Aires, Argentina

## Abstract

In view of its ability to explain the most frequent motor symptoms of Parkinson’s Disease (PD), degeneration of dopaminergic neurons has been considered one of the disease’s main pathophysiological features. Several studies have shown that neurodegeneration also affects noradrenergic, serotoninergic, cholinergic and other monoaminergic neuronal populations. In this work, the characteristics of cholinergic deficits in PD and their clinical correlates are reviewed. Important neurophysiological processes at the root of several motor and cognitive functions remit to cholinergic neurotransmission at the synaptic, pathway, and circuital levels. The bulk of evidence highlights the link between cholinergic alterations and PD motor symptoms, gait dysfunction, levodopa-induced dyskinesias, cognitive deterioration, psychosis, sleep abnormalities, autonomic dysfunction, and altered olfactory function. The pathophysiology of these symptoms is related to alteration of the cholinergic tone in the striatum and/or to degeneration of cholinergic nuclei, most importantly the nucleus basalis magnocellularis and the pedunculopontine nucleus. Several results suggest the clinical usefulness of antimuscarinic drugs for treating PD motor symptoms and of inhibitors of the enzyme acetylcholinesterase for the treatment of dementia. Data also suggest that these inhibitors and pedunculopontine nucleus deep-brain stimulation might also be effective in preventing falls. Finally, several drugs acting on nicotinic receptors have proved efficacious for treating levodopa-induced dyskinesias and cognitive impairment and as neuroprotective agents in PD animal models. Results in human patients are still lacking.

## Introduction

Parkinson’s disease (PD) is a progressive neurodegenerative disorder affecting about 1 person out of every 1,000 in their fifth decade and 19 out of every 1,000 in their eighth decade or older.^1^ Its principal epiphenomenological clinical symptoms are abnormal involuntary movements, bradykinesia, rigidity, and tremor. Patients also frequently display non-motor symptoms, including cognitive impairment, mood disorders, sleep alterations, dysautonomia, and hallucinations, among others.^2^

Histopathological changes are mainly, but not exclusively, characterized by the progressive loss of the nigrostriatal dopaminergic pathway and of the source dopaminergic neurons in the substantia nigra pars compacta, which explain the most typical motor symptoms.^3^ Administration of levodopa to parkinsonian patients has been considered the most effective symptomatic treatment for the last 40 years.^4^

At a cellular level, neuronal death may be preceded by a series of dysfunctional states, including loss of redox control, alteration of lysosomal activity, abnormal protein control mechanisms in the endoplasmic reticulum (ER) and perturbation of the ER–Golgi trafficking mechanisms. These cellular pathologies are closely intertwined with one of the hallmarks of the disease, namely the abnormal accumulation of misfolded protein aggregates.^5^ Lewy bodies constitute a characteristic pathological finding, second only to the neurofibrillary tangles in Alzheimer's disease (AD). Early work identified the immunoreactivity of the Lewy bodies with antibodies against the presynaptic protein α-synuclein.^6^ One major target of α-synuclein is Rab1, a key component of the ER–Golgi trafficking pathway.^7^ ER stress has been invoked as a possible major disruptive mechanism, leading to an adaptive reaction known as the unfolded protein response.^8^ This response may be cytoprotective when activated to a moderate level, but is deleterious at a higher level, triggering in turn the apoptotic death of the damaged neuron.^9,10^ PD may also be considered a synaptopathy, i.e., abnormal synaptic connectivity compromising nigrostriatal pathways and intrastriatal interneuronal connections, presumably most apparent at the initial stages of the disease. Mutations in the α-synuclein gene cause familial forms of PD and dementia with Lewy bodies. Synaptic accumulation of α-synuclein is accompanied by the redistribution of the synaptic SNARE proteins SNAP-25, syntaxin-1, and synaptobrevin-2, as well as by an age-dependent reduction in dopamine release.^11^

The striatum is the most important input nucleus of the basal ganglia. The principal source of afferents is layer 5 of the cerebral cortex, conveying glutamatergic (Glu) excitatory synapses. Motor areas (4 and 6 plus supplementary motor area) together with the primary somatosensory cortex follow, also with Glu neurotransmission. The second major striatal input is dopaminergic (DA), stemming from the substantia nigra A9 cell group. There are many features of PD that are unresponsive to levodopa, such as gait disorders and cognitive impairment or dementia, indicating the involvement of other neurotransmitter systems;^12^ in this regard, recent evidence suggests degeneration of adrenergic, serotoninergic, and cholinergic neurons, among others.^12^

The importance of cholinergic dysfunction in the physiopathology of many PD features cannot be overemphasized. For example, in a recent study in 137 PD patients, cholinergic denervation could be related to rapid eye movement (REM) behavior disorder, fall history, gait disorders, and cognitive dysfunction.^13^ Furthermore, antagonists of the muscarinic acetylcholine (ACh) receptors (AChRs), derived from *Atropa belladonna*, were used to treat akinetorigid disorders in the nineteenth century,^14^ long before the formal recognition of PD and the development of modern treatment strategies. In this review, recent evidence concerning the extent of cholinergic denervation in PD and its clinical correlates are discussed. A summary of cholinergic neurotransmission in health will precede the sections on cholinergic deficits in PD.

Bibliographical references were searched in Pubmed by the following string: (acetylcholine OR cholinergic) AND PD. Articles in English, Spanish, or French were retrieved. Reference sections from retrieved papers were also explored for database enrichment.

## Cholinergic neurotransmission in health

**Figure 1:**
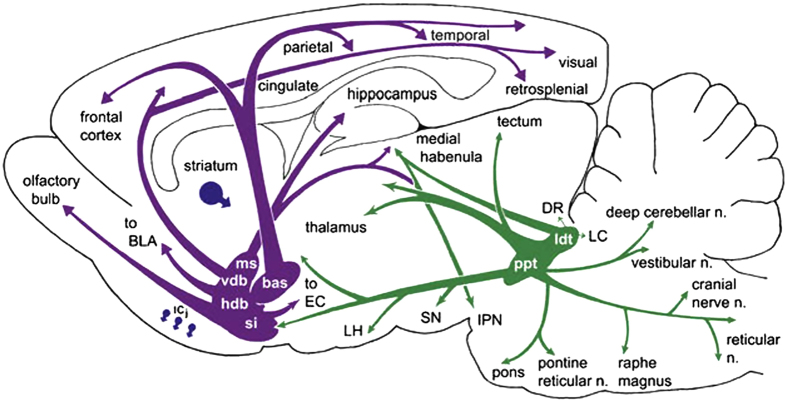
Cholinergic neurons and networks in the rodent CNS. bas, nucleus basalis; BLA, basolateral amygdala; DR, dorsal raphe; EC, entorhinal cortex; hdb, horizontal diagonal band nucleus; Icj, islands of Cajella; IPN, interpeduncular nucleus; LC; locus ceruleus; ldt, laterodorsal tegmental nucleus; LH, lateral hypothalamus; ms, medial septal nucleus; PPN, pedunculopontine nucleus; si, substantia innominata; SN, substantia nigra; vdb, vertical diagonal band nucleus. Reprinted from Woolf and Butcher, Cholinergic systems mediate action from movement to higher consciousness, 2011, with permission from Elsevier.

ACh is an ubiquitous, small molecular weight neurotransmitter which has a pivotal role in chemical neurotransmission in the central (CNS) and peripheral nervous system (PNS). In the brain, ACh mediates distant signaling through projection neurons and local signaling via interneurons; the type of message conveyed by ACh depends on a variety of factors, including site of release, the localization of the target neurons, the target receptor subtypes,^15^ and the status of the target cells at the time of release. Furthermore, ACh signaling may be circumscribed to the synapse or result from the delocalized diffusion of the neurotransmitter in the extracellular milieu and binding to nonsynaptic sites.^16,17^ In terms of gross anatomical brain regions it is safe to state that ACh affects brain in its entirety. Figure 1 schematically summarizes the cholinergic nuclei and connectivity in the rodent CNS. One can clearly appreciate that cholinergic reach spans subcortical as well as cortical domains. A recent review^18^ meticulously dissects current knowledge on the anatomy of cholinergic projections, summarized in two main tracks: (i) the brainstem- and (ii) the magnocellular basal forebrain-cholinergic systems. The former, as worked out by immunochemical techniques,^19^ involves neuronal soma located in the pedunculopontine tegmental nucleus (PPN) and the laterodorsal pontine tegmentum (LDT) and projecting to the thalamus, basal ganglia, the basal forebrain and to a much lesser extent, the cerebral cortex. The basal forebrain cholinergic system comprises neurons located in the medial septal nucleus (MS), the vertical and horizontal limbs of the diagonal band of Broca, and the nucleus basalis magnocellularis (NBM, the nucleus basalis of Meynert in humans), which send projections to neocortex, entorhinal cortex, limbic cortices, cingulate cortex, and hippocampus. Cholinergic fibers in cortex appear not to be associated with postsynaptic densities, a fact that has been linked with the hypothesis that cholinergic transmission may not be synaptic but may involve modulation of target neurons via diffusion, so-called volume transmission.^16^

From the standpoint of the target receptors, ACh neurotransmission is mediated through two entirely different types of receptor proteins and ensuing molecular mechanisms, i.e., the metabotropic seven transmembrane domain muscarinic AChRs and the ionotropic pentameric nicotinic acetylcholine receptors (nAChRs). The former are members of the G-protein-coupled superfamily of receptors, which possess seven transmembrane segments and mediate intracellular signals associated with metabolic cascades. The nAChRs, on the other hand, are members of the superfamily of pentameric ligand-gated ion channels, a collection of neurotransmitter receptors which also includes γ-amino butyric acid (GABA-A, GABA-C), glycine, serotonin (5-HT3), and bacterial homologs.^20,21^

The metabotropic muscarinic acetylcholine receptors (mAChRs) are coupled to different types of G proteins, e.g., G_i/o_ type (M2 and M4 subtypes of mAChRs) of G proteins that negatively couple to adenylate cyclase or G_q_ proteins (M1, M3, and M5 subtypes of mAChRs), which convert the cholinergic signal into metabolic cascades.^22^ Presynaptic mAChRs (M2, M4 subtypes) are largely inhibitory and perform this function partly as inhibitory autoreceptors on cholinergic terminals.^23^ The M2 subtype is the predominant autoreceptor in the hippocampus and cerebral cortex, whereas M4 is the main subtype in the striatum.^15,22,24^ Postsynaptic mAChRs can be either inhibitory (M2, M4) or excitatory (M1, M3, M5).^15,24^

The ionotropic, fast-signaling nAChRs are composed of five polypeptide subunits organized pseudosymmetrically around a central pore.^25^ Each subunit contains an extracellular domain, four hydrophobic transmembrane segments arranged in the form of three concentric rings around the pore and a short extracellular carboxy-terminal domain. nAChRs are characteristically involved in the rapid “phasic” effects of ACh under conditions of brief release/high local concentration of the neurotransmitter, but they also operate under the low, tonic ACh release or mimicking systemically applied cholinergic drugs,^16^ a condition that may be particularly relevant to cholinergic neurotransmission—and/or its modulation—in the striatum.

Muscle-type nAChRs are expressed in the PNS and neuronal-type nAChRs in both PNS and the CNS as well as in other non-neural tissues such as immune cells, lymphocytes, lung epithelium, and other tissues.^26^ In the CNS, the nAChR is present in various combinations of subunits (α4, α5, α6, α7, α9, α10, and β2),^27^ the two most abundant ones being the heteropentameric receptor formed by α4 and β2 subunits and the homopentameric receptor formed exclusively by α7 subunits. The deficit of some of the nAChR subunits in PD has been explicitly explored.^28^ The two predominant forms of the nAChR, the α4β2 and the α7 oligomers, are also strongly expressed in the striatum, accompanied by the α6β2 form.^29^ It is not clear whether other subunits are present in the heteromeric nAChRs. The subcellular localization of the α4β2 and α6β2 forms in the striatum is the dopaminergic terminal, the predominant target undergoing degeneration in PD. The α4β2 nAChR is also found in striatal GABAergic inhibitory interneurons.^30^

The α7 nAChR exhibits certain functional properties that distinguish it from other nicotinic receptors: (a) fast desensitization kinetics; (b) unusually high Ca^2+^ permeability; and (c) high affinity for binding α-bungarotoxin.^27,31^ In most regions of brain the α7 nAChR is found presynaptically, where it modulates enhanced neurotransmitter release of various other neurotransmitters, including dopamine, 5-HT, glutamate, and GABA, and postsynaptically, where it generates postsynaptic currents.^32,33^ In addition, the perisynaptic presence of the receptor has also been demonstrated, where it modulates neuronal activity, presumably by an unconventional mechanism involving diffusion of the natural neurotransmitter and binding to non-synaptic sites.^16^ In the striatum, the α7 nAChRs are found in cortical glutamatergic excitatory afferents.^32^

Cholinergic mechanisms are intimately linked to cognitive functions associated with cortical and hippocampal brain anatomical regions. Working memory, spatial and episodic memory acquisition storage, maintenance and retrieval, attention, and other neurophysiological processes at the root of neural information and cognitive functions remit to ACh neurotransmission at synaptic, pathway, and circuital levels.

## Cholinergic disturbances in PD

A summary of the brain cholinergic nuclei exhibiting signs of denervation in PD and their clinical correlates is presented in Table 1. Whenever possible, throughout the rest of the paper, each subsection corresponding to a feature or symptom will contain a brief introduction followed by data on animal models, then on PD patients and finally on treatment strategies.

### Motor function

#### Introduction

The dorsal striatum, receiving the input from the substantia nigra dopaminergic neurons, is mainly accountable for regulating the cognitive aspect of motor learning and the subsequent actual realization of the motor functions. The SN-striatal projections are the phylogenetically best conserved of all connections across tetrapods, from amphibians to mammals. Among brain structures, the striatum contains some of the highest levels of ACh and DA. The two neurotransmitter systems interact extensively in a bidirectional manner, both at the presynaptic and postsynaptic levels, mediating cognitive mechanisms, the selection of motor responses, and reward.^34,35^ The cholinergic interneurons, key actors in this interplay,^34,35^ represent about 1% of the total population of striatal neurons.^36^ The cholinergic interneurons are giant cells whose soma can reach up to 40 μm in diameter, and which do not possess spines (“aspiny”). Their physiological role has been extensively reviewed recently.^36–39^ Here only fundamental aspects of this complex function will be mentioned. It should be noted firstly that striatal cholinergic interneurons (SCIs), although scarce in absolute numbers, send projections widely throughout the striatum, establishing synaptic contacts over a vast striatal territory;^40^ SCIs fire tonically at a rate of 3–10 Hz, as a result of which there is a pulsatile release of ACh under resting conditions,^41^ which does not produce receptor desensitization, probably due to the counteracting high acetylcholinesterase activity in the striatum. It has been observed that changes in this cholinergic tone may contribute to associative learning, particularly the relationship between environmental cues and outcomes. Input to these interneurons include glutamate (coming from the thalamic caudal intralaminar nuclei), GABA, substance P and enkephalin (coming from striatal medium spiny neurons), among others.

#### Role of acetylcholine receptors

As previously mentioned, both nicotinic and muscarinic AChRs are found in the striatum. Several pieces of evidence suggest that ACh release can trigger dopamine release from the nigrostriatal varicosities by acting on nAChRs, whereas activation of the mAChRs can produce the opposite effect. The metabotropic mAChRs have either facilitatory action (via the M1-subtype mAChRs) or inhibitory action (via M2-subtype mAChRs). These receptors are located presynaptically on corticostriatal and nigrostriatal afferents, or exhibit a somatodendritic distribution on the medium spiny neurons. Furthermore, DA appears to inhibit SCIs, which are therefore overactive in PD. In turn, this causes subsequent inhibition of dopamine release by muscarinic activation, thus creating a vicious cycle. It should be mentioned that there is no direct contact between dopaminergic and cholinergic neurons, underlining the important role of extracellular DA, which is known to be released from nigrostriatal terminals in a tonic, rhythmic pacemaker fashion under basal conditions.^42^ The tonic release results in a basal local concentration in the striatum in the low nanomolar range, which can shift to the micro- and millimolar range upon activation of midbrain dopaminergic neurons.^42^ These bursts of DA activity mute the cholinergic interneuron activity. In addition, SCI also regulates the activity of medium spiny neurons, modifying excitability to cortical or nigral inputs.

#### Cholinergic control of striatal output

The control of striatal GABAergic circuits by choline acetyltransferase (ChAT)-positive cholinergic interneurons has been suggested to offer strong integrative opportunities based on networks of interneurons that participate not only in movement but also in attention and reinforcement-related mechanisms.^43^ Only one striatal GABAergic interneuron, the so-called neuropeptide-Y expressing neurogliaform neuron, had not been identified until recently.^44,45^ It has recently been discovered that in addition to the slow GABAergic interneurons, a novel class of fast-adapting GABAergic interneurons operates under the control of neostriatal cholinergic interneurons through fast nAChR-mediated synaptic inputs.^43^ In the hippocampus, α7 nAChRs are concentrated in spiny interneurons in association with calcium-ATPase pump isoform 2 and establish critical links with the scaffold postsynaptic protein PSD-95.^46^

#### Treatment of PD motor symptoms by anticholinergic drugs

Anticholinergics were the first drugs available for the symptomatic treatment of PD and they are still widely used today, both as monotherapy and as part of combination regimes. Their efficacy has been assessed by many randomized controlled trials, and their results were summarized in a systematic review and meta-analysis in the early 2000’s.^14^ Nine double-blind cross-over placebo-controlled studies including 221 patients were identified, in which the effects of benzhexol, orphenadrine, benztropine, bornaprine, benapryzine, and methixine were assessed. All studies except one (dealing with methixine) found a significant improvement from baseline with the anticholinergic drug in at least one outcome measure. There was no evidence that any of the drugs was more beneficial for tremor than for other motor symptoms. Neuropsychiatric and cognitive adverse events were a frequent cause of drop-out. Anticholinergics have been considered as ‘clinically useful’ drugs in monotherapy or in combination with levodopa for the treatment of motor symptoms in PD by the most recent Movement Disorder Society Evidence-Based Medicine Review.^47^

### Gait impairments

#### Introduction

Gait impairments, disturbed balance and falls are among the most relevant determinants of an impaired quality of life and increased mortality among PD patients.^48,49^ The PPN appear to be key players in motor coordination.^50^ There is some evidence indicating that gait impairments and falls in PD are correlated with dysfunction of the cholinergic PPN.^51^

#### Data from studies on animal models

Animal model studies have yielded some conflicting results. On the one hand, they underline the importance of PPN degeneration in the search for gait impairments in PD. Effects of PPN lesions on gait parameters in 10 MPTP-treated young or aged monkeys were assessed in a recent study.^52^ PPN were lesioned either with a toxin specific for cholinergic neurons or with a nonspecific one. Interestingly, hypokinesia, and tremor improved after lesion with the specific cholinergic toxin. However, both the specific of the nonspecific toxin impaired gait and worsened postural parameters. Apomorphine reversed gait deficits after MPTP, but not after PPN lesions. These results suggest that PPN regulates movement by two distinct mechanisms, one of which may be connected with the basal ganglia. Effects of PPN stimulation were also explored in 6OHDA-lesioned rats.^53^ Automated gait analysis was employed to assess stride length, base of support, and the maximum area of a paw that came into contact with the glass plate. The base of support and maximum area were larger in lesioned animals, and this was partially corrected by PPN stimulation.

In contrast to the results described in the preceding paragraph, some reports have challenged the importance of PPN degeneration. Results from a study in rats found that gait was altered when cholinergic neurons from the basal forebrain were lesioned, was maximal when dopaminergic nigrostriatal neurons were also lesioned, and was independent of the functional status of the PPN.^54^ The authors suggested that basal forebrain cholinergic–striatal disruption of attentional-motor interactions may have been responsible for the findings.

#### Data from studies in PD patients

Studies conducted in PD patients suggest an effect of cholinergic denervation at the PPN and NBM in gait disturbances. In a study involving 17 PD with history of falls, 27 PD non-fallers and 15 healthy controls, 1-[^11^C]methylpiperidin-4-yl propionate ([^11^C]PMP) position emission tomography (PET) imaging was used to evaluate cholinergic terminal integrity by measuring the presence of the enzyme acetylcholinesterase (AChE).^55^ Cortical activity represented NBM integrity and thalamic uptake reflected PPN integrity. In addition, (+)-(^11^)C-dihydrotetrabenazine ([^11^C]DTBZ) vesicular monoamine transporter type 2 (VMAT2) brain PET imaging was used to assess dopaminergic input to the striatum. PD patients showed lower levels of AChE and VMAT binding compared with controls. AChE levels were lower in PD fallers compared with non-fallers, without differences in VMAT. These findings suggest the involvement of both cholinergic systems in falls, which are otherwise independent of dopaminergic degeneration.

In a subsequent trial, gait parameters were correlated with cholinergic and dopaminergic denervation in 125 nondemented PD patients and 32 controls.^56^ Cortical cholinergic denervation was heterogeneous, with 38 subjects with PD (30.4%) exhibiting cortical AChE activity below normal range and 21 subjects (16.8%) with PPN-thalamic AChE activity below normal range. Gait speed was found to be lower in the group exhibiting cortical AChE activity below normal range, but displayed no relationship with PPN-thalamic AChE activity. One of the limitations of this study, as acknowledged by the authors, was that measurement of gait speed could not distinguish subjects with hesitations and freezing from those walking slowly for other reasons. Therefore, in a subsequent study, PD patients without Freezing of Gait (FoG) were studied by using the same techniques for mapping brain cholinergic denervation.^57^ In this study, the proportion of patients with freezing was greater among those with neocortical AChE below normal range and with neocortical amiloidopathy. Conversely, no relationship with PPN–thalamic activity was found. Interestingly, a significant trend effect was seen, with FoG frequency being lowest with absence of both pathological conditions, intermediate in subjects with single extranigral pathological condition and highest with combined neocortical cholinopathy and amyloidopathy. In a cross-sectional study including more than 600 PD patients, exposure to anticholinergics was also related to FoG.^49^

Finally, it should be mentioned that reduced cholinergic activity in the PPN, but not in the neocortex, was observed in PD patients considered as unsafe vehicle drivers, compared with safe drivers.^58^ Interestingly, dopaminergic innervation did not differ in these groups. These results reinforce the notion that PPN might integrate sensory input with motor responses. The authors found surprising that neocortical cholinergic activity, related to cognitive process, was not altered in risky drivers. Notwithstanding, these results should be interpreted cautiously due to a reduced sample size and lack of cognitive assessment.

#### Therapeutic potential of cholinergic manipulations

The evidence of the involvement of cholinergic nuclei in gait disorders led to the suggestion that interfering with its activity might somehow lead to beneficial effects on gait. The efficacy and safety of unilateral PPN deep brain stimulation (PPN DBS) in PD was explored by means of a double-blinded evaluation with intraoperative neurophysiological and postoperative imaging characterization of the surgical target.^59^ Six PD patients with severe off-period gait and balance impairment and freezing with falls causing severe limitations to their ability to carry out the normal activities of their daily life, despite optimization of medical treatment, were included. Clinical double-blind trials were performed 3 and 12 months after surgery, assigning patients to ON or OFF stimulation. There were no major objective or subjective motor differences, including in gait parameters, in the ON versus OFF conditions at 3 or 12 months post surgery. However, patients reported less falls under both ON and OFF conditions after surgery (UPDRS Item 13—falling—at baseline: 2.0±09, at month 3: 0.5±0.5, and at month 12: 0.5±0.5, both *P*<0.05 versus baseline). These results are compatible with the evidence from imaging studies, where PPN-thalamic cholinergic denervation was related to falls but not to FoG or other gait parameters. It should be noted, however, that in addition to the caveat of the small size of the sample the authors state other possible explanations for these results, such as low instrumental sensitivity, insertion with neural disruption-related changes in function, a placebo component and the prolonged maintenance of benefit after cessation of chronic stimulation.^59^ Importantly, although the procedure was not accompanied by adverse events.

In another attempt to improve gait and reduce falls by manipulating cholinergic pathways, donepezil (a centrally acting inhibitor of AChE) was administered to 23 subjects with PD who reported falling or nearly falling more than 2 times per week.^60^ The study was designed as a randomized, placebo-controlled, crossover trial, with treatment phases of 6 weeks and a 3-week wash-out period in between. Interestingly, fall frequency per day on placebo was 0.25±0.08 compared with 0.13±0.03 on donepezil (*P*<0.05).

#### Summary

The bulk of evidence suggests that gait disturbances and falls in PD may be related to cholinergic loss in the NBM and/or PPN. The mechanisms are unclear, but may involve attention deficits and altered motor coordination.^61^ More research in animal models might help further clarify this issue and reconcile conflicting results. Drugs enhancing cholinergic tone might effectively reduce falls, but effects on other gait parameters are not known. Evidence of the potential efficacy of PPN-DBS is still controversial, and thus no recommendation can be made at the present time. Furthermore, drugs blocking muscarinic receptors might have deleterious effects in these domains and caution should thus be employed in their application in PD patients.

### Levodopa-induced dyskinesias

#### Introduction

Levodopa-induced dyskinesias (LIDs) are choreoathetosic hyperkinetic movements that complicate motor treatment with dopaminergic drugs in PD and have profound effects on the quality of life.^62^ Their physiopathology is complex and involves changes in neurotransmitters and gene expression,^63^ including cholinergic abnormalities.

As mentioned earlier, cholinergic tone induces dopamine and glutamate release by activating presynaptic nAChRs on dopaminergic and glutamatergic terminals.^32,64^ Activation of mAChRs and nAChRs also contributes to plasticity of glutamatergic and GABAergic inputs to the striatum.^65,66^ Upon nigrostriatal denervation, ACh release is increased,^67^ which in turn facilitates long-term potentiation (LTP).^68^ LTP has been associated with dyskinetic behavior in animal models of PD.^69^ Therefore, increased cholinergic tone might be one of the factors leading to LIDs in PD. In the last few years, compelling evidence supporting this concept has emerged.

#### Data from studies on animal models

The role of cholinergic striatal interneurons in LIDs has been recently explored in a study with 6OHDA-lesioned or Pitx3-deficient *aphakia* mice (*Pitx3*-^ak/ak^), which show nigrostriatal dopaminergic deficits.^70^ One of the outcomes of the study was the expression extracellular signal-regulated kinase1/2 (ERK), which is thought to mediate the expression of LIDs. In both animal models, acute dopamine challenge induced ERK activation in medium spiny neurons in the denervated striatum, whereas diminished activation in medium spiny neurons and increased activity in striatal cholinergic interneurons were observed after repeated levodopa administration. Interestingly, pharmacological blockers of ERK and a muscarinic antagonist (dicyclomine) reduced LIDs. The authors hypothesized that increased sensitivity of cholinergic neurons to dopamine might contribute to LIDs and that antagonizing cholinergic tone might help reduce them. These results have been confirmed recently by one study showing that selectively inducing cholinergic cell death reduced LIDs.^71^ Interestingly, this manipulation did not interfere with the improvement of motor symptoms following levodopa administration.

Given the richness of the striatum in nAChRs, especially, the α4β2, α6β2, and α7 subtypes,^27,31,72^ the effects of nicotine on LIDs were explored in the MPTP monkey PD model.^73^ Nicotine was uptitrated to 650 mcg/ml and levodopa challenges were conducted during the maintenance phase. Animals were then crossed over to placebo. Nicotine significantly reduced the severity of LIDs. No effects were observed in the antiparkinsonian effects of levodopa. Similar results with nicotine were observed in rats and mice,^74,75^ and also when some nicotinic agonists, such as TC-8831,^76^ ABT-089 and ABT-894,^77^ ABT-107,^78^ and AQW051 were tested.^79^

The mechanism by which nicotine might reduce LIDs was further explored in a study on 6OHDA-lesioned rats.^75^ Interestingly, in addition to the already known antidyskinetic effect of nicotine, mecamylamine, a nicotinic receptor blocker, also reduced LIDs. Both nicotine and mecamylamine had effects only when applied chronically and their effects were not additive. Altogether with the findings that nicotine or mecamylamine treatments both resulted in declines in α6β2 nAChRs in the unlesioned, but not in the lesioned striatum, these results led the authors to suggest that nicotine might act via desensitization of its receptors, i.e., reduction in channel activity and/or receptor internalization induced by the agonist.^80^ In this model, nicotine or nAChR antagonists would reduce LIDs by preventing the action of endogenous ACh on their target nAChRs.

The next logical step was to explore the efficacy of nicotine on LIDs and this was actually undertaken in an experiment in the MPTP-treated monkey PD model.^81^ Nicotine (300 mcg/ml) was administered in drinking water (over 4–6 months) to levodopa-primed or levodopa-naive monkeys. Results showed that nicotine pretreatment and post-treatment were similarly efficacious in reducing LIDs.

The question of which subtypes of nAChRs were involved was tackled in a series of experiments.^29,72,80^ In α4β2 and α6β2 knockout mice, reduced baseline LID levels and lack of nicotine effect were observed, reinforcing the concept that nAChRs are necessary for both the occurrence of LIDs and the antidyskinetic effect of nicotine. Similar studies showed that baseline LIDs were actually higher in α7 nAChR null mutant mice, whereas nicotine treatment still reduced LIDs. It was interpreted that α7 nAChRs exerted an inhibitory control on the occurrence of LIDs.

#### Data from studies in PD patients

Clinical evidence on the role of anticholinergic agents in LIDs is scanty and controversial, some with positive and some with negative results.^39^ In a study conducted by Pourcher *et al.*,^82^ nine patients with LIDs went through an acute levodopa challenge. All patients underwent a challenge with only levodopa and another one with levodopa plus etybenzatropine, a muscarinic antagonist. No change in the delay of action of levodopa or in the percentage of improvement of parkinsonism was observed when etybenzatropine was administered. The absolute duration of onset and end-of-dose dyskinesias tended to be shorter with either drugs, but this failed to reach statistical significance. The maximal severity of onset and end-of-dose dyskinesia was slightly but significantly lessened with etybenzatropine. On the other hand, there are some case reports of patients developing dyskinesias after treatments with anticholinergics.^83^

#### Summary

There is robust evidence indicating the important role of nAChRs in the genesis of LIDs. The good results yielded by many compounds in preclinical PD models warrants their application in clinical trials. Clinical evidence is scanty and controversial. The role of muscarinic receptors in LIDs still remains to be studied.

### Cognitive impairment and mood disorders

#### Introduction

The physiopathology of PD dementia (PDD) is complex and involves severe dopaminergic and cholinergic deficits, the main pathological drivers of cognitive decline being a synergistic effect between α-synuclein and Alzheimer's disease’s (AD) pathology.^84^ Only cholinergic deficits will be reviewed in this section.

Because of their distribution in brain anatomical regions associated with cognitive processes, various subtypes of nAChR have been invoked as being associated with abnormal cognitive processes. The α7 nAChR is highly expressed in the hippocampus, a region particularly affected in cognitive disorders,^27,85–87^ as recently reviewed in ref. 88, whereas a massive loss in cerebral cortex of the other most abundant type of CNS nAChRs, the α4β2-type, accompanies the cognitive decline observed in AD.^89,90^ Of course alterations in memory and cognition associated with nAChRs have also been reported in pathological states other than AD, such as schizophrenia.^91^ The various functions afflicted in PD have been associated with nAChR dysfunction of different brain nAChR oligomeric forms.^92–94^ α7 nAChR ligands are a subject of intense research in diseases affecting cognitive functions, especially the subclass of ligands termed positive allosteric modulators (PAMs; see reviews in ref. 95), a group of compounds that enhance recognition memory and cognitive improvement in animal models (e.g., refs 96, 97).

Muscarinic receptors are also implicated in cognitive disturbances. Antagonists such as scopolamine perturb the performance of cognitive tasks in animal models^98^ and even lead to extreme cognitive disturbances with delirium at higher doses.^99^ This condition has also been reported in children after application of postsurgical transdermal patches to ameliorate nausea and motion sickness^100^ and in the elderly, who are particularly vulnerable to even modest levels of antimuscarinic drugs due to their cumulative effects.^101^

Several lines of evidence link brain nicotinic nAChRs, the α7 in particular, with the development of neurodegenerative disease with cognitive impairments, like AD.^95^ The greater the magnitude of depletion of cholinergic neurons and associated cholinergic pathways in cognitive-associated brain areas such as the neocortex and hippocampus, the more severe the associated dementia, suggesting a relationship between the clinical manifestations and the level of cholinergic decline.^102,103^ Cholinergic pathways are associated with the processes of learning and memory, and nicotinic agonists and cholinomimetics in general have been used as therapeutic agents providing symptomatic improvements in cognitive impairment.^104–108^ This constitutes the basis of therapeutic approaches aiming at α7 AChR activation with selective agonists.

#### Data from studies in PD patients

The involvement of ACh pathways in PD is exemplified by the results of a recent trial by Park *et al*.^109^ White matter hyperintensities in the cholinergic pathways were assessed by means of the Cholinergic Pathways Hyperintensities Scale (CHIPS) after 3.0-tesla magnetic resonance. Patients with AD (*n*=20), PDD (*n*=21) and Dementia with Lewy Bodies (DLB, *n*=17) were compared with a group of 20 healthy controls. Results showed that the CHIPS score was correlated with MMSE, SOB scores of the Clinical Dementia Rating, and verbal and visuospatial memory domains in demented patients.

Degeneration of the NBM appears to be highly correlated with PDD.^110^ A recent study showed that PD patients with mild cognitive impairments (PD-MCI) who would develop PDD during follow-up had greater degeneration of the substantia innominata, where the NBM is located.^111^ In this study, 51 PD-MCI were followed for a minimum of 2 years, during which PDD was diagnosed in 15 cases. Greater grey matter loss in the prefrontal area was also observed in subjects developing PDD. Loss of neurons in the substantia innominata was observed in early stages of the disease, and was further accentuated in PDD.

Recent results in post-mortem analyses of brains from demented and non-demented PD patients confirmed these results. In the study by Hall and colleagues, stereological analyses of the A9 and A10 dopaminergic neurons and Ch1, Ch2, and Ch4 cholinergic neurons located in the basal forebrain, along with an assessment of α-synuclein pathology in these regions and in the hippocampus, were performed in six demented and five non-demented PD patients and five age-matched control individuals with no signs of neurological disease.^112^ ChAT activity in the hippocampus and frontal cortex was also measured in a different set of eight demented and eight non-demented PD patients, as well as in the same areas of eight age-matched controls. Stereological analyses showed a significant 54% reduction in the NMB of PDD compared with controls and a non-significant reduction of 30% in non-demented PD. No differences were observed in other cholinergic regions. Furthermore, the density of ACh neurons in the NBM correlated inversely with the severity of dementia. ChAT activity, a measure of the presence of cholinergic terminals in a given brain region, was reduced in the hippocampus of PD with dementia compared to non-demented patients and controls. Interestingly, neocortical ChAT activity was reduced in the neocortex of both demented and non-demented PD compared with controls. Finally, α-synuclein pathology and Lewy-body deposition in the basal forebrain of patients with PDD were more severe than in non-demented patients, thus suggesting the possible role of α-synuclein aggregation in the development of cortical and hippocampal cholinergic dysfunction.

The density of the α4β2 nAChR in the CNS has been recently correlated with cognitive impairments in non-demented PD patients.^113^ Previous studies have revealed reduced binding to these receptors in PD brains, and some preliminary findings suggest that the density of these receptors might correlate with cognitive impairments. In this study, 25 non-demented PD patients underwent a 5-[123I]iodo-3-[2(*S*)-2-azetidinylmethoxy]pyridine (5-I-A-85380) SPECT to visualize α4β2 nAChRs and cognitive testing with the CERAD (Consortium to Establish a Registry for Alzheimer’s Disease) battery to identify domains of cognitive dysfunction.^113^ Results showed significant correlations between performance of the CERAD subtests Boston Naming Test (a specific test for visual perception and for detection of word-finding difficulties) and Word List Intrusions (a specific test for learning capacity and memory for language information) with the density of α4β2 nAChRs at the right superior parietal lobe cortex and the left thalamus, and left and right posterior subcortical regions.

An interesting question is whether the alteration of the NBM is the same as that found in AD. NBM degeneration is comparable or even more intense in PD compared with the latter, yet the clinical characteristics of the two dementias differ significantly. Some authors have suggested that the divergence may be connected to possible differences in the degree to which subsections of the NBM are affected,^114^ but this hypothesis remains to be studied.

Depression can precede dementia, or at least depressed patients are at greater risk of developing PDD.^115^ In a recent study, neocortical cholinergic innervation was assessed in 12 non-demented PD patients, 6 PDD, and 10 normal controls^116^ by means of dynamic PET scanning of previously injected [^11^C]methyl-4-piperidinyl propionate radioligand, a selective substrate for AChE hydrolysis. Pooled analyses demonstrated a significant inverse correlation between cortical AChE activity and Cornell Scale for Depression in Dementia scores (*r*=0.5, *P*=0.007). The correlation remained significant when only PD patients were assessed, in whom AChE activity also correlated with the MMSE score. Recent evidence suggests that the early involvement of the posterior neocortex and visuoperceptual impairment may be risk factors for the rapid symptomatic progression and dementia in PD.^117^

#### Cognitive dysfunction as a side-effect of cholinergic drugs

In the light of the evidence reviewed above, it is not surprising that drugs interfering with cholinergic function have profound effects on cognitive function in PD. Muscarinic receptor blockers can cause acute confusion, dementia, and chronic intellectual impairment.^61^ In a study with trihexyphenidyl, an oral anticholinergic agent, clinical disability, cognitive assessment, and measurements of cerebral blood flow (rCBF) and oxygen metabolic rate (rCMRO) were performed in six PD before and after administration of trihexyphenidyl for 7 weeks at 6 mg/day.^118^ Results showed improvements in motor symptoms without evident changes in cognitive function. Cortical and striatal rCBF and rCMRO2 were significantly decreased, a typical finding in PDD.^119^

#### Therapeutic potential of cholinergic manipulations

At the other end of the spectrum, inhibitors of the enzyme cholinesterase can improve cognition in PD. A recent systematic review and meta-analysis suggested that AChE inhibitors are effective in the treatment of cognitive impairment in patients with PD.^120^ The systematic search yielded three studies involving donepezil and one involving rivastigmine. Results showed that these drugs significantly slowed MMSE decline (MD=−1.123, 95% confidence interval (CI)=−1.638 to −0.608; *P*=0.001; *I* ^2^=44.6%), and ADAS-cog (SMD=−0.266, 95% CI −0.399 to −0.133; *P*<0.0001; *I* ^2^=0%). Interestingly, the death rate was reduced in treated patients as compared with those receiving a placebo (OR=0.295, 95% CI 0.108–0.806; *P*=0.017; *I* ^2^=0%). In a separate analysis for rivastigmine and donepezil, the results were confirmed for cognitive function. Tremor and adverse drug reactions in general were more frequent with cholinesterase inhibitors, but no differences were observed in the risk of falling. Rivastigmine is considered to be “Clinically Useful” for the treatment of dementia in PD according to the latest review of the Movement Disorder Society Evidence-Based Medicine Task Force.^121^

#### Summary

Robust evidence indicates that PDD is related to loss of cholinergic input to neocortical structures. Furthermore, drugs enhancing cholinergic neurotransmission appear to be effective in improving cognitive function and those blocking muscarinic receptor seem to increase the risk of cognitive impairment in PD.

### Psychosis and delirium

#### Introduction

Visual hallucinations (VHs) are frequently reported in some forms of PD associated with dementias such as LBD.^122^ Besides the effect of dopaminergic medication, anticholinergics are associated with VHs even in patients without PD.

#### Data from studies in PD patients

In a recent study, inhibitory cholinergic activity in the CNS was measured by means of the short-latency afferent inhibition (SAI) technique in 10 non-demented PD patients with VHs, in 12 non-demented PD patients without VHs and in 11 age-matched healthy controls.^123^ Results showed reduced SAI in patients with VHs, which was otherwise normal in patients without hallucinations. In addition, patients with VHs showed more frequent MCI and had reduced values in some cognitive function tests. The authors speculated that these results might be related to reduced neocortical cholinergic input from the NBM.

#### Delirium

Characterized by an acute and fluctuating disturbance in attention and awareness accompanied by an additional disturbance in cognition, delirium is more frequent in PD than in the general population.^124^ Cholinergic deficiency is one of the most frequently found abnormalities in delirium. There are no studies specifically targeting this symptom in PD.

#### Summary

VHs in PD and perhaps also delirium are related to cholinergic dysfunction. However, further studies are required to clarify this relationship.

### Sleep disturbances

#### Introduction

Sleep disturbances are common disabling non-motor features of PD that have a detrimental effect on health-related quality of life.^122^ Activation of the PPN is capable of inducing REM sleep^125^ and degeneration of cholinergic neurons in the basal forebrain and brainstem is one of the factors resulting in a reduction in REM sleep and REM-sleep Behavior Disorder (RBD).^126^ RBD is characterized by a loss of normal muscle atonia during REM sleep and dream-enacting behavior. RBD occurs in 0.5% of the general population, but is considered as a risk factor for synucleinopathies and even as a premotor sign of PD.^127^

#### Data from studies on animal models

The effects on REM sleep of drugs acting on different monoaminergic systems have been explored in the MPTP mouse model of PD.^128^ The objective of the study was to assess the effects of these drugs on sleep/wakefulness patterns, measuring the amount of REM sleep (or paradoxical sleep—PS). Arecoline, a muscarinic agonist, increased the amount of PS in the MPTP-treated mice but not in the controls, probably reflecting supersensitivity in the former.

#### Data from studies in PD patients

Cholinergic function has been recently evaluated in PD patients with or without RBD by means of the short latency afferent inhibition (SAI), a transcranial magnetic stimulation protocol able to test an inhibitory cholinergic circuit in the human brain.^129^ In this study, 10 PD patients with RBD diagnosis by polysomnography, 13 patients without the disorder and 10 healthy controls were enrolled. In addition to SAI, neuropsychological examination was also performed. SAI was reduced in PD patients with RBD compared with unaffected PD and healthy controls. Interestingly, MCI was more frequent in the former, and cognitive parameters correlated with SAI. These findings indicate that cholinergic dysfunction may have an important role in RBD in PD.

#### Therapeutic potential of cholinergic manipulations

The results in MPTP monkeys reviewed earlier suggest that drugs enhancing cholinergic activity might help improve RBD, but no data are available in PD. Notwithstanding, RBD symptoms were improved by rivastigmine in two small case series of patients with DLB.^130^ Further results in PD are awaited.

#### Summary

Cholinergic degeneration is likely involved in the genesis of RBD in PD. Cholinesterase inhibitors might be effective for the treatment of this disorder, though further evidence on this is required.

### Olfactory dysfunction

#### Introduction

Olfactory dysfunction affects ~90% of PD patients.^131^ Interestingly, such dysfunction is observed in the premotor phase of the disease, when dopaminergic degeneration is not yet evident. Therefore, damage to largely non-dopaminergic neurotransmitter systems may contribute to, or possibly even cause, the olfactory loss observed in PD and some other neurodegenerative diseases. A large body of evidence supports the involvement of cholinergic neurotransmission in olfactory dysfunction.

#### Data from studies on animal models

A recent study in MPTP-treated monkey showed less ChAT density in the inner layers of the OB as compared with controls.^132^ Cholinergic denervation was also observed in the horizontal limb of the diagonal band of Broca, in the basal forebrain, the nucleus that gives rise to cholinergic centrifugal projections to the OB.

Olfactory function was further explored in Prp-A53T-α-synuclein transgenic (αSynA53T) mice, which had been reported to show age-dependent motor impairments and cytoplasmic inclusions, and in wild controls.^133^ Cholinergic and dopaminergic systems in the OB of mice were explored by immunofluorescence staining, enzyme linked immunosorbent assay and western blot. Results showed that αSynA53T mice displayed a deficit of odor discrimination and odor detection starting at the age of 6 months, which was accompanied by a marked decrease in cholinergic neurons and a decrease in AChE activity in the OB.

#### Data from studies in PD patients

A study on 24 functionally anosmic, non-demented PD patients, 39 non-anosmic, non-demented PD, and 29 healthy controls showed that cognitive impairment, the result of cholinergic pathway degeneration as discussed earlier, correlates with olfactory dysfunction.^134^ The possibility that these results could be explained by cholinergic degeneration affecting both domains was further explored in subsequent studies. Odor identification, cognitive functioning, and cholinergic degeneration (as assessed by [^11^C]PMP PET imaging) were explored in 58 non-demented PD patients.^135,136^ Results showed that impaired odor identification correlated on the one hand with cognitive impairment and on the other with AChE activity in the limbic region (hippocampus and amygdala).

Furthermore, in a recent study, cholinergic innervation of the olfactory bulb (OB) was explored in brains from patients with AD, PD and DLB and compared with healthy controls.^132^ PD and AD patients showed less CHAT density in the inner layers of the OB as compared with controls. Density was even lower in DLB.

#### Summary

Olfactory dysfunction appears to correlate with cholinergic deficits in the OB. Such deficits are also related to α-synuclein pathology.

### Peripheral organs functioning

#### Introduction

Cholinergic neurons provide parasympathetic autonomic innervation to peripheral organs as well as sympathetic innervation to the skin. Cholinergic dysfunction might thus contribute to alterations in innervated organs in PD.

#### Data from studies on animal models

Cholinergic alterations in the dorsal motor nucleus of the vagus (DMV), origin of parasympathetic afferents controlling gut function, were correlated with gastrointestinal dysfunction in the 6OHDA-lesioned rat.^137^ Results showed a reduced number of ChAT-positive neurons in the DMV and slowing down of the gastric transit.

#### Data from studies in PD patients

Cholinergic innervation to peripheral organs was explored in 12 non-demented early-to-moderate PD patients and 12 healthy controls.^138^ 5-[^11^C]-methoxy-donepezil PET was used to assess the integrity of cholinergic function in the body of the studied subjects and information on motor severity, constipation, gastroparesis, and other parameters was also collected. Heart rate variability measurements and gastric emptying scintigraphies were performed in all subjects to obtain objective measures of parasympathetic function. Significant decreased ^11^C-donepezil uptake was observed in the small intestine, pancreas, and myocardium. No correlations were found between the ^11^C-donepezil signal and disease duration, severity of constipation, gastric emptying time, and heart rate variability. The authors hypothesized that such results reflected parasympathetic denervation, as it was previously shown that no loss of intestinal cholinergic neurons occurred and that α-synuclein pathology is not located in those neurons. Results involving the pancreas are intriguing and may explain data connecting PD with diabetes and glucose intolerance.^139^ However, the low sample size may have obscured other differences.

Changes in perspiration, which is under the control of the cholinergic sympathetic system, are frequent in PD.^122^ Using the sympathetic skin response (SSR) in the palms to electrically stimulate the median nerve at the wrist, Schestasky showed diminished response in PD patients as compared with controls.^140^ In another study, α-synuclein deposition and the density of intraepidermal, sudomotor, and pilomotor nerve fibers were measured in skin biopsies of PD patients and healthy controls.^141^ PD patients showed distal sensory and autonomic neuropathy characterized by loss of intraepidermal and pilomotor fibers with greater α-synuclein deposition.

#### Summary

Reduced cholinergic tone to peripheral organs due to neuronal degeneration is frequent in PD and might explain the occurrence of many non-motor symptoms, including constipation, perspiration, and increased risk of diabetes, among others. ACh is an important parasympathetic effector in the bladder, and antimuscarinic drugs are effective for urge incontinence in PD.^142^ However, to the best of our knowledge there is no study specifically addressing cholinergic neurotransmission to the bladder and urinary tract.

### Neuroprotection

#### Introduction

Neuroprotection is probably the most important unmet need in PD.^143^ Cigarette smoking that is otherwise a well-known health hazard and one of the leading avoidable causes of morbidity and mortality, has been shown to reduce the risk of PD in numerous case-report and cohort studies.^144^ This apparent neuroprotective effect is correlated with increased intensity and duration of smoking, is reduced with smoking cessation, and occurs with different types of tobacco products.

#### Data from studies on animal models

Several pieces of evidence coming from studies on different parkinsonian animal models indicate that nicotine exposure improves dopaminergic markers and function in the lesioned striatum, the brain region predominantly affected in PD.^145^ These effects have been linked to the activation of nAChRs.^146^

In a recent paper, the effects of nicotine and α7-type neuronal nAChRs on H_2_O_2_-induced astrocyte apoptosis and glial cell-derived neurotrophic factor (GDNF) downregulation were studied.^147^ The effects showed that nicotine inhibited H_2_O_2_-induced astrocyte apoptosis in a dose-dependent manner. Furthermore, nicotine reduced motor deficits in MPTP-treated mice. Similarly, nicotine was able to prevent the reduction of GDNF induced by H_2_O_2_. Interestingly, antagonists of the α7 nAChRs were able to block all the aforementioned effects of nicotine. The authors hypothesized that the underlying mechanisms might involve alleviation of mitochondrial membrane potential loss, stabilization of the Bax/Bcl-2 balance and inhibition of cleaved caspase-9 activity through the activation of the α7 nAChRs.

In another series of experiments the neuroprotective effects of pretreatment with ABT-107, an α7 nAChR agonist, were tested in 6OHDA-lesioned rats.^148^ Pretreatment with nicotine, which was used as a positive control, reduced motor deficits. These effects were also observed with ABT-107. The drug was able to reduce dopaminergic neuronal loss in the striatum and increased basal dopamine release.

#### Summary

Nicotine exhibits consistent neuroprotective effects in animal studies, which may be promising in PD. These effects appear to be mediated, at least in part, by the α7 nAChRs, and one agonist of these receptors produced similar effects. Therefore, studies in patients are warranted.

## Discussion

The cholinergic neurotransmission system is severely affected in PD, with widespread denervation. Compelling evidence links these abnormalities to major clinical features of PD, namely, motor symptoms, gait dysfunction, levodopa-induced dyskinesias, cognitive deterioration, psychosis, sleep abnormalities, autonomic dysfunction, and altered olfactory function. The neuroprotective properties of nicotine and nAChR agonists also suggest that cholinergic degeneration may contribute to the degeneration of dopaminergic neurons. The results reviewed in this article have deep impact on the clinical prognosis and management of PD.

Recent results support the notion of different PD “subtypes”, clinically characterized by specific combinations of symptoms. For example, several studies report clinical differences between patients with or without dominant tremor.^149,150^ In the latter, PD progresses more rapidly, akinesia is more severe and other features such as cognitive impairment, psychosis, or mood disorders are more frequent.^151^ This group is characterized by more severe dopaminergic depletion, but the plethora of symptoms is evocative of cholinergic denervation. To the best of our knowledge this has never been put to the test.

The multitude and magnitude of cholinergic pathologies in PD highlights the likely importance of drugs increasing or decreasing cholinergic tone. On the one hand, muscarinic antagonists, administered in low doses to avoid adverse atropinic side effects, are clinically useful drugs for the treatment of motor symptoms.^47^ On the other hand, increasing cholinergic tone by administering AChE inhibitors is an efficacious strategy for improving PDD,^121^ reducing fall rate,^60^ and probably also psychosis and RBD; however, well-conducted clinical trials in support of the latter claims are lacking. Notwithstanding, it should be acknowledged that effects are typically modest. It is seldom the case that a drug is effective in such a large number of relevant clinical domains, and AChE inhibitors could thus acquire considerable importance in the management of PD in the near future. However, these potential benefits need to be weighed against the risk of confusional states and hallucinations, which are not infrequently observed with cholinergic drugs.

The search for neuroprotective therapies has become a major objective in the PD research agenda. So far, no drug has offered convincing evidence of disease-modifying effects.^143^ Guided by the observation that smokers have reduced risk of developing PD, several drugs acting on nicotinic receptors have been tested in PD animal models, with promising results. Results in human patients are still to come.

In conclusion, cholinergic dysfunction is frequent and severe in PD. Clinically, dysfunction is connected with motor symptoms, gait dysfunction, levodopa-induced dyskinesias, cognitive impairment, RBD, psychosis and olfactory loss, among others. This suggests that drugs modifying the cholinergic tone might be front-line agents for the management of PD. Results with potentially neuroprotective drugs acting on nicotinic receptors are encouraging and clinical trials are awaited.

## Figures and Tables

**Table 1:** Sources of cholinergic dysfunction in PD and its main clinical correlates

*PD feature*	*Pathological basis*	*Possible treatment*
PD motor symptoms	Altered cholinergic striatal tone	Antimuscarinic drugs in low doses to avoid atropinic side effects
Gait impairment and falls	Degeneration of the NBM and/or the PPN nuclei	To reduce falls: AChE inhibitors and possibly PPN DBS (controversial)
Levodopa-induced dyskinesias	Altered cholinergic striatal tone	Drugs acting on nicotinic receptors (in pre-clinical stages)
Cognitive Impairment	Degeneration of the NBM	AChE inhibitors (proven efficacy)
RBD	Degeneration of the PPN	AChE inhibitors (never tested)
Psychosis	Reduced cholinergic tone (site unknown)	AChE inhibitors (never tested)
Neuroprotection	Mechanism unknown	Drugs acting on nicotinic receptors (in preclinical stages)

Abbreviations: AChE, Acetylcholinesterase enzyme; DBS, deep brain stimulation; NBM, nucleus basalis magnocellularis (Meynert’s nucleus); PD, Parkinson’s disease; PPN, pedunculopontine nucleus; REM, rapid eye movement; RBD, REM-sleep Behavior Disorder.
